# Insulin sensitivity affects propensity to obesity in an ethnic-specific manner: results from two controlled weight loss intervention studies

**DOI:** 10.1186/1743-7075-10-3

**Published:** 2013-01-08

**Authors:** Barbara A Gower, Jessica A Alvarez, Nikki C Bush, Gary R Hunter

**Affiliations:** 1 Department of Nutrition Sciences, University of Alabama at Birmingham, Birmingham, AL, USA; 2 Department of Human Studies, University of Alabama at Birmingham, Birmingham, AL, 35294, USA; 3 Current address: Division of Endocrinology, Diabetes & Lipids, Emory University School of Medicine, Atlanta, GA, 30322, USA; 4 Current address: Endocrine Research Unit, Mayo Clinic, Rochester, MN, 55905, USA; 5 Department of Nutrition Sciences, University of Alabama at Birmingham, 423 Webb Building, 1675 University Blvd, Birmingham, AL, 35294-3360, USA

**Keywords:** Glycemic index, Diet, Insulin secretion, Acute insulin response

## Abstract

**Background:**

Risk for obesity differs with ethnicity/race and is associated with insulin sensitivity (S_I_), insulin responsiveness, and dietary glycemic load (GL). The objective of this study was to test the hypotheses that, 1) obesity-prone, normal weight, African-American (AA) women would be more insulin sensitive than BMI-matched, never overweight AA women; 2) increased adiposity over time would be associated with greater baseline S_I_ and higher dietary GL in AA but not European-American (EA) women; and 3) increased adiposity over time would be predicted by S_I_ in women with high but not low acute insulin response to glucose (AIRg).

**Methods:**

Two controlled weight loss interventions were conducted involving overweight (BMI 25.0-29.9 kg/m^2^) premenopausal AA and EA women. The first included matching with normal-weight (BMI <25.0 kg/m^2^) controls following weight loss, and then comparing S_I_. The second included a 1-year follow-up of weight-reduced participants to identify predictors of change in %body fat. Main outcome measure in the first study was insulin sensitivity (S_I_) as assessed with intravenous glucose tolerance test (IVGTT), and in the second study was change in %fat, as assessed with DXA, over one year. AIRg was assessed during IVGTT, and free-living diet was determined by food record.

**Results:**

In the first study, formerly overweight AA women were 43% more insulin sensitive than BMI-matched never overweight AA (*P* < 0.05). In the second study, S_I_ was positively associated with change in %fat over 1 year only in AA women (*P* < 0.05) and women with high AIRg (*P* < 0.05). In addition, AA who were insulin sensitive and who consumed a higher GL diet tended to gain greater %fat (*P* = 0.086 for diet x S_I_ interaction). In both studies, AA women had higher AIRg (*P* < 0.001) than EA women.

**Conclusions:**

Formerly overweight (obesity-prone) AA women were more insulin sensitive than never overweight AA women, a quality that may predispose to adiposity, particularly when combined with a high GL diet. This ethnicity/race-specific effect may be due to high insulin responsiveness among AA.

## Background

Risk for obesity is disproportionately high among African-American (AA) women. U.S. epidemiological data indicate that the age-adjusted rate of overweight and obesity is 82% in AA women, and the prevalence of grade 3 obesity is higher in AA women (18%) than in all other race/gender sub-groups [1]. The reason for this disparity is not clear, but may relate to inherent differences in metabolic factors, in particular, insulin responsiveness. Numerous studies have shown that healthy AA relative to EA have up to 2-fold greater insulin response [2-6]. This higher insulin response has been attributed to greater insulin secretion and/or lower clearance, and is independent of differences in insulin sensitivity [2-4,7].

There are several ways through which insulin may promote adiposity. Insulin has profound effects on both carbohydrate and lipid metabolism [8]. Its actions on glucose uptake promote glycogen synthesis and glucose oxidation. Its lipogenic and anti-lipolytic effects promote triglyceride storage. Thus, it seems plausible that insulin may promote adipose tissue accrual via these processes. In addition, insulin may affect hunger/satiety and thereby food intake via central or peripheral actions [9,10]. However, the effect of insulin on voluntary food intake in humans is complex, and neither its actions nor their mechanisms have been entirely elucidated [11].

Because insulin secretion is stimulated by glucose ingestion, dietary carbohydrate (CHO) quantity and/or quality may interact with insulin secretion to influence adiposity. In support of this possibility, interactive effects of insulin secretion with diet on weight gain have been documented in both humans and animal models. Within a large population of healthy free-living women and men, 6-year weight gain was positively associated with insulin concentrations at 30 and 60 min following ingestion of oral glucose [12]. The relationships were significant only among individuals consuming a higher CHO, lower fat, diet. In rats, an interaction between diet and indices of glucose metabolism regarding weight gain has been reported [13]. Analogous data have been reported with weight loss, where greater weight loss occurred in conjunction with a low glycemic load (GL) diet within individuals with a high insulin response at baseline [14].

Further, the effects of insulin on physiological processes may be enhanced by greater sensitivity to insulin. Several reports have indicated that insulin sensitivity predicts weight gain. Individuals who were more insulin sensitive at baseline gained more weight or %fat [15-17] over a given follow-up period. Given their relative hyperinsulinemia, AA may be particularly sensitive to the effects of both diet and insulin sensitivity on risk for obesity.

The objective of the study was to test the hypotheses that, 1) obesity-prone, normal weight, African-American (AA) women would be more insulin sensitive than BMI-matched, never overweight AA women; 2) increased adiposity over time would be associated with greater baseline S_I_ and higher dietary GL in AA but not European-American (EA) women; and 3) increased adiposity over time would be predicted by S_I_ in women with high but not low acute insulin response to glucose (AIRg). To address these hypotheses, we examined data from two controlled diet intervention studies involving overweight women. The first involved matching weight-reduced women with normal-weight (BMI <25.0 kg/m^2^) controls to determine if obesity propensity affected insulin sensitivity. The second involved a 1-year follow-up of weight-reduced participants to determine if insulin response, insulin sensitivity, and dietary GL predicted weight gain.

## Methods

### Study 1: Participants and study design

The data used for this study were from an intervention project designed to determine if overweight/obese women are physiologically different from never-overweight, lean control women. Details and main outcomes of the study have been published [18-20]. In brief, we have reported that weight loss has favorable effects on metabolic risk factors in this cohort of women [18]; that energy expenditure does not differ in weight-reduced women and lean controls [20]; and that gain in adiposity in the combined cohort was related to respiratory quotient [19]. In none of these previous reports did we determine whether insulin sensitivity differed in weight-reduced women vs lean controls, and whether ethnicity/race affected this comparison; this is the objective of the current study.

**Figure 1:**
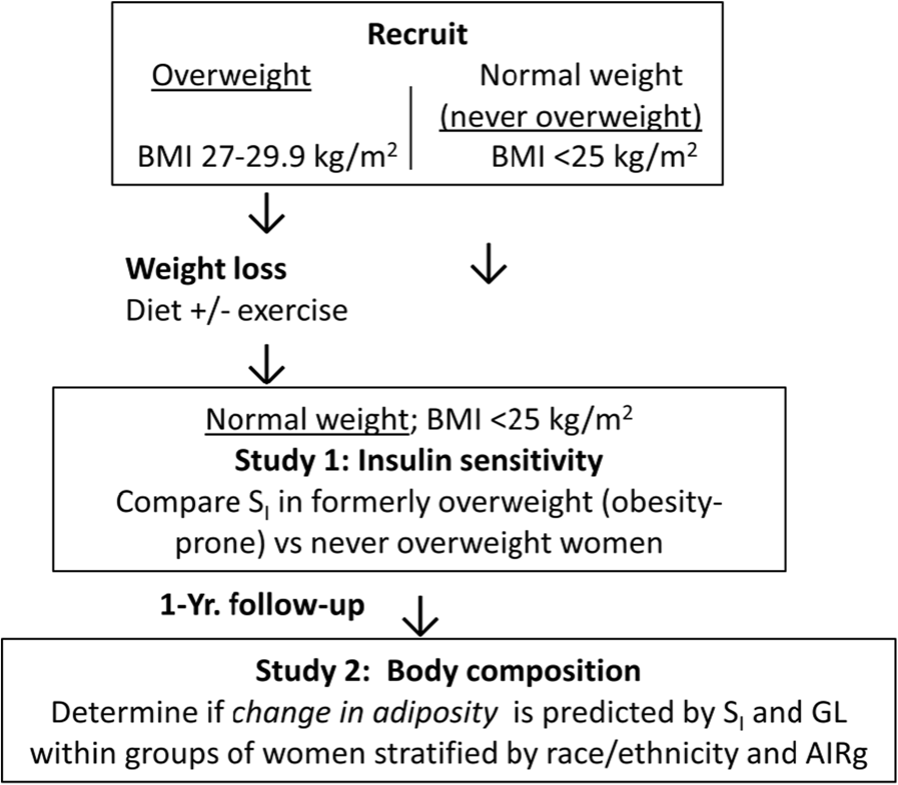
**Schematic of experimental design for the two weight loss intervention studies.** For **Study 1**, overweight women were provided with a controlled weight loss intervention (low-energy diet) until they achieved BMI <25 kg/m^2^, at which time insulin sensitivity was assessed and compared to a group of never overweight women of BMI < 25 kg/m^2^. For **Study 2**, overweight women were provided with a controlled weight loss intervention (diet +/− exercise) until they achieved BMI <25 kg/m^2^, at which time insulin sensitivity was assessed (“baseline”). Women were evaluated for change in body composition after 1 year.

Participants were 82 healthy, sedentary (≤1 day/week structured exercise), pre-menopausal women with (*n* = 35) or without (*n* = 47) a personal and family history of obesity. Overweight women were recruited at a BMI of 25–29.9 kg/m^2^, and underwent diet-induced weight loss as described [18]. These women were evaluated after reducing their BMI to <25 kg/m^2^, immediately following a 4-week period of supervised weight stability (Figure 1). The weight loss program involved consuming a low-energy diet (800 kcal/d) until target BMI was reached (approximately 16 weeks). Fifty women started the weight loss program. Of the 50 women who started the weight loss program, 35 both completed the program and had a successful post-weight-loss insulin sensitivity test (i.e., a test not compromised by failed iv or hemolyzed blood samples). Data from these 35 women were used. At the same time as the weight loss intervention, a never overweight “control” group (*n* = 50) was recruited that had a similar ethnic composition (50% EA and 50% AA) and age as the starting group of obesity-prone women, and whose BMI was within the target range of the obesity-prone group (20–25 kg/m^2^). The never overweight group had no personal or family history of obesity. No participant used medications that affect body composition or metabolism. All were nonsmokers and reported experiencing menses at regular intervals. Prior to testing, all participants were placed on a weight-maintenance diet for 4 weeks. At the end of the 4-week weight-maintenance period, body composition, fat distribution, and metabolic outcomes were assessed under controlled conditions during an in-patient stay at the General Clinical Research Center (GCRC). All testing was conducted within 12 days of menses.

### Study 2: Participants and study design

The data used for this study were from an intervention project designed to determine if aerobic or resistance training was more effective in maintaining weight loss in a group of overweight/obese AA and EA women. Details and main outcomes of the study have been published [21-23]. We also reported that in the combined group of women, greater insulin sensitivity predicted greater gain in %body fat over 1 year; that greater glucose disposal predicted less gain in intra-abdominal fat; and that dietary glycemic load interacted with insulin sensitivity to predict gain in adiposity [17]. In this earlier study, the data were not examined separately by ethnicity/race. Thus, the objective of this study was to determine if ethnicity/race differences existed in the association of insulin sensitivity with changes in adiposity, and if these associations were mediated by postchallenge insulin response.

Participants were 103 healthy overweight (BMI 25–29.9 kg/m^2^) pre-menopausal women with a family history of overweight in at least one first-degree relative. Women underwent a controlled period of weight loss by diet alone or diet in combination with exercise as previously described [24,25]. None used medications that affect body composition or metabolism. All were nonsmokers and reported experiencing menses at regular intervals. For the purposes of this study, “baseline” was taken as post weight loss, at which time all participants had a BMI of <25 kg/m^2^ (Figure 1). Insulin sensitivity data were not available on several participants due to failed iv or hemolyzed blood samples. Thus, data were used from only those participants having insulin sensitivity information.

Prior to baseline testing, all participants were placed on a weight-maintenance diet for 4 weeks. At the end of the 4-week weight-maintenance period, body composition and metabolic outcomes were assessed under controlled conditions during an in-patient stay at the GCRC. All testing was conducted within 12 days of menses. Women then entered a 1-year follow-up period where they were encouraged to maintain their reduced weight status and were offered nutrition education classes. Those women who were assigned to the exercise arms of the weight loss intervention also had access to a gym facility during the follow up period, and were encouraged to continue to exercise. Preliminary analyses indicated that results from women prescribed nutrition education plus exercise did not differ from those of women prescribed nutrition education without exercise. Thus, a “group” variable was not included in the final analyses. At the conclusion of the 1-year follow-up period, body composition was assessed.

Studies were approved by the Institutional Review Board for Human Use at the University of Alabama at Birmingham (UAB). All women provided informed consent before participating.

### Body composition

Body composition was measured by dual-energy X-ray absorptiometry (DXA) in the Department of Nutrition Sciences at UAB. A Lunar DPX-L (software version 1.35, November 1997; GE-Lunar, Madison, WI) was used in the first study, whereas a Lunar Prodigy densitometer and enCORE software version 6.10.029 (2002) was used in the second study. Participants were scanned in light clothing while lying flat on their backs with arms at their sides.

### Insulin sensitivity

Insulin sensitivity was assessed on an in-patient basis in the GCRC after an overnight fast with a tolbutamide-modified (first study) or an insulin-modified (second study) frequently-sampled intravenous glucose tolerance test (IVGTT). Prior to testing, flexible intravenous catheters were placed in the antecubital spaces of both arms. Three, 2.0 ml blood samples were taken over a 20-min period for determination of basal glucose and insulin (the average of the values was used for basal "fasting" concentrations). At time "0", glucose (50% dextrose; 11.4 g/m^2^) was administered intravenously. Tolbutamide (125 mg/m^2^) or insulin (0.02 U/kg, Humulin, Eli Lilly and Co., Indianapolis) was injected at 20 min post glucose injection. Blood samples (2.0 ml) were collected at the following times (min) relative to glucose administration: 2, 3, 4, 5, 6, 8, 10, 12, 15, 19, 20, 21, 22, 24, 26, 28, 30, 35, 40, 45, 50, 55, 60, 70, 80, 100, 120, 140, 180.

Sera were stored at −85°C until analyzed. Glucose and insulin values were entered into the MINMOD computer program (ver. 3, © Richard N. Bergman) for determination of the insulin sensitivity index (S_I_) [26]. The acute insulin response to glucose (AIRg) was calculated as the incremental insulin area-under-the-curve from minutes 0–10 following glucose injection using the trapezoidal method.

### Diet

For the second study, information on habitual diet was collected using 4-d food records. Participants were asked to complete the records at the 1-year time point, prior to the 2 weeks of food provision and metabolic evaluation. Instructions regarding completion of the record were provided in person by a registered dietitian. A handout that reiterated the instructions and contained information regarding portion size also was provided. Participants were directed to provide brands of food items, location of meal consumption (e.g., cafeteria, restaurant, home), and method of cooking (e.g., fried, broiled), and to specify the type of fat used in cooking. A dietitian reviewed each record upon completion, and contacted participants to clarify any questions. Food records were analyzed by a registered dietitian using the Nutrition Data System for Research (NDS-R) software (Nutrition Coordinating Center, University of Minnesota, MN, version 2007). Only food records with at least 3 of the 4 days completed were used for analysis, and the days were averaged for mean nutrient intake. Because not all women returned the records, dietary information was available on 68 women. Mean daily dietary glycemic load, a measure that reflects both carbohydrate quantity and quality [27], was used as an independent variable in statistical analysis.

### Laboratory analyses

All analyses were conducted in the Core Laboratory of UAB’s GCRC, Diabetes Research and Training Center (DRTC), and Nutrition Obesity Research Center (NORC). Glucose was measured using an Ektachem DT II System (Johnson and Johnson Clinical Diagnostics, Rochester, NY). In the Core laboratory, this analysis has a mean intra-assay CV of 0.61%, and a mean inter-assay CV of 2.56%. Insulin was assayed in duplicate using Diagnostic Products Corporation (Los Angeles, CA) "Coat-A-Count" kits (first study) or Linco Research Inc. double-antibody RIA (St. Charles, MO, second study). In the Core Laboratory, these assays have a sensitivity of 1.9 μIU/ml, a mean intra-assay CV of 5%, and a mean inter-assay CV of 6% (DPC), and a sensitivity of 3.35 μIU/ml, a mean intra-assay CV of 3.49%, and a mean inter-assay CV of 5.57% (Linco).

### Statistical analysis

All statistical analyses were performed using SAS (version 9.2; SAS Institute, Inc., Cary, NC). Fasting insulin, AIRg, and S_I_ were log10 transformed prior to statistical analysis. All statistical tests were two-sided and were performed using a Type I error rate of 0.05.

For the first study, main effects of obesity status [formerly overweight (obesity-prone) vs never overweight], ethnicity/race, and the obesity status x ethnicity/race interaction on participant characteristics and main outcome variables including S_I_, were determined by ANOVA using unadjusted data. 2-way ANCOVA (adjusting for %fat) was subsequently used to further examine the main effects of ethnicity/race and obesity status, and the obesity status x ethnicity/race interaction, on S_I_.

For the second study, between-group differences in participant characteristics at baseline and 1 year were determined using ANOVA. Pearson correlation analysis was used to examine the associations of dietary GL with fasting insulin and AIRg within each ethnic/race group. To examine independent and interactive effects of insulin phenotype and dietary GL with change in %fat, participants were divided into groups based on median categories of S_I_ and GL (high/low for each variable). Two-way ANOVA was conducted within each ethnic/race group for the dependent variable change in %fat, examining main effects of GL, S_I_, and the GL x S_I_ interaction. Because all significant observations occurred within the AA women, additional analyses were conducted to determine if greater AIRg in AA could potentially explain the unique association of S_I_ with change in % fat in this group. These analyses were conducted by ANCOVA (adjusting for GL) within AA and EA separately, and within participants with high and low AIRg separately (based on median AIRg).

## Results

Participant characteristics for the first study are shown in Table 1 by obesity status [formerly overweight (obesity-prone), never overweight] and ethnicity/race. Formerly overweight women on average were older, weighed more, and had a higher BMI. EA women had higher S_I_.

**Table 1:** Objective 1: characteristics of the study population (mean ± SEM) by obesity status [formerly overweight (obesity-prone) or never overweight] and ethnicity/race

	**Formerly overweight**		**Never overweight**		
	**EA**	**AA**	**EA**	**AA**	Main effects*
Age (yr)	38.4 ± 1.3	36.4 ± 1.2	31.4 ± 1.1	31.8 ± 1.1	Obesity
Body weight (kg)	66.3 ± 1.4	64.4 ± 1.3	62.8 ± 1.1	60.3 ± 1.2	Obesity
BMI (kg/m^2^)	24.3 ± 0.3	23.9 ± 0.3	23.0 ± 0.2	22.6 ± 0.2	Obesity
Body fat (%)	34.0 ± 1.1	33.6 ± 1.1	32.5 ± 1.0	31.6 ± 1.0	
Fasting Insulin (μIU/ml)	7.5 ± 0.7	7.3 ± 0.7	7.4 ± 0.6	7.0 ± 0.6	
S_I_ [x10^-4^ min^-1^/(μIU/ml)]	5.94 ± 0.86	5.48 ± 0.77	7.91 ± 0.95	4.09 ± 0.53	Race, Ob x Race
AIRg (μIU/ml x 10 min)	357 ± 67	506 ± 92	305 ± 47	536 ± 87	Race

*Significant (*P* < 0.05) main effects of obesity status (Obesity), ethnicity/race (Race), or the Obesity by race/ethnicity (Ob x R) interaction by ANOVA; unadjusted data.

Abbreviations: EA = European-American; AA = African-American; BMI = body mass index; S_I_ = insulin sensitivity index; AIRg = acute insulin response to glucose.

**Figure 2:**
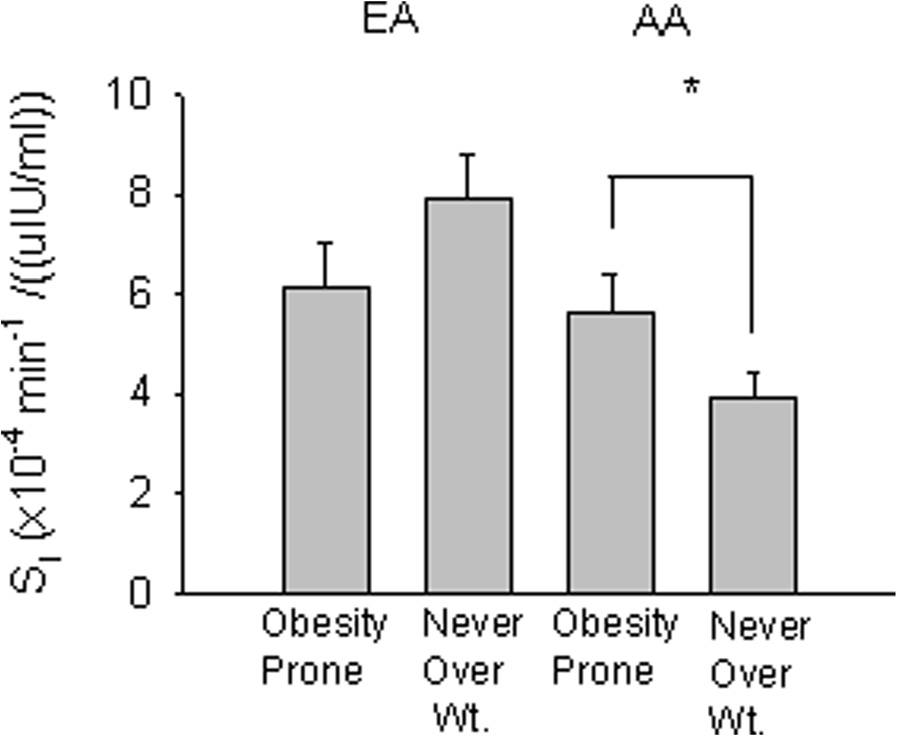
**Insulin sensitivity by ethnic group and obesity status.** Formerly overweight (obesity-prone) AA women were more insulin sensitive than never overweight AA women (*P < 0.05), whereas obesity-prone EA women did not differ from never overweight EA women (*P* = 0.158; adjusted for %fat). *P* < 0.05 for the group x ethnicity/race interaction.

Analysis of covariance for S_I_ by obesity status [formerly overweight (obesity-prone), never overweight] and ethnicity/race revealed a significant main effect of ethnicity/race (lower among AA; *P* < 0.01), and a significant obesity status x ethnicity/race interaction (*P* < 0.05, data adjusted for %fat). Formerly overweight (obesity-prone) AA women were 43% more insulin sensitive than never overweight AA women (*P* < 0.05). In contrast, formerly overweight (obesity-prone) EA women were 22% less insulin sensitive than never overweight EA women (Figure 2).

Participant characteristics for the second study are shown in Table 2A by ethnic/race group. At baseline, all women were BMI <25 kg/m^2^ (weight reduced, formerly overweight). Ethnic/race composition of the participants was 44% European-American and 56% African-American. AA had lower S_I_, lower fasting glucose, and higher AIRg. The 68 women with available dietary intake data did not differ in any way from the group as a whole, being similar with respect to age, BMI, ethnic/race distribution, and insulin sensitivity [17].

**Table 2:** Objective 2: characteristics of study population (mean ± SEM) by ethnic/race group at baseline (A) and at 1 year (B)

**A. Baseline**	**EA (*n *= 46)**	**AA (*n *= ;57)**
Age (yr)	34.7 ± 0.9	34.5 ± 0.8
Body weight (kg)	65.8 ± 0.9	64.9 ± 0.8
Body mass index (kg/m^2^)	23.7 ± 0.2	23.9 ± 0.1
Body fat (%)	34.1 ± 0.7	32.3 ± 0.6*
Fasting glucose (mg/dL)	87 ± 1	83 ± 1**
Fasting insulin (μIU/ml)	8 ± 3	8 ± 3
Insulin sensitivity [S_I_; x 10^-4^ min^-1^/(μIU/ml)]	4.73 ± 0.28	3.76 ± 0.25**
Acute insulin response to glucose (AIRg; μIU/ml x 10 min)	416 ± 65	794 ± 59***
**B. 1 year.**	EA	AA
Energy intake (kcal/d)^1^	1399 ± 52	1281 ± 49
Carbohydrate intake (g/d) ^1^	177 ± 9	157 ± 9
Protein intake (g/d) ^1^	63 ± 3	54 ± 3*
Fat intake (g/d) ^1^	51 ± 3	50 ± 3
Glycemic load^1^	99 ± 5	91 ± 5
Δ%fat	+4.8 ± 0.4	+5.7 ± 0.4
Δ Lean mass (kg)	−0.09 ± 0.23	−0.30 ± 0.20

*P < 0.05, **P < 0.01, ***P < 0.001 for EA vs AA.

^1^For diet data: *n* = 31 EA; *n* = 36 AA.

Abbreviations: EA = European-American; AA = African-American.

Dietary information at 1 year, and changes in outcomes of interest after 1 year, are shown in Table 2B. EA women consumed more protein (g/d) than AA women. Reported intake of total energy, carbohydrate, and fat did not differ with ethnic group. Total energy intake averaged ~1300 kcal/d, suggesting that the women under-reported their intake, as previously documented [28]. Pearson correlation analysis indicated that dietary GL was associated with fasting serum insulin concentration among AA (*r* = 0.48, *P* < 0.01) but not among EA (*r* = −0.04, *P* = 0.853). GL was not associated with AIRg in either group (AA: *r* = −0.17, *P* = 0.379; EA: *r* = −0.12, *P* = 0.535).

**Figure 3:**
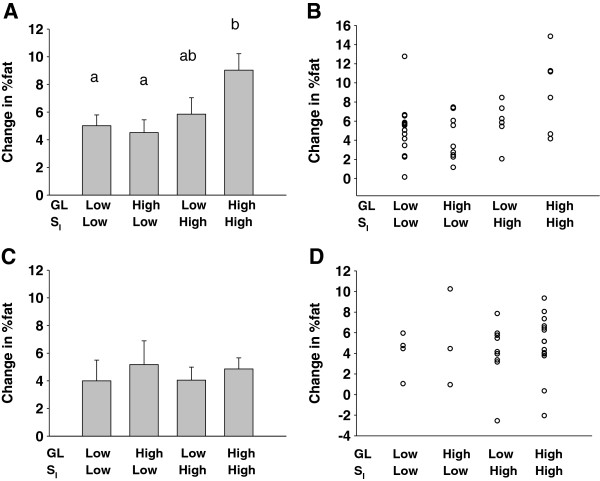
**Change in %fat over 1 year by median glycemic load (GL) and median insulin sensitivity (S**_**I**_**) in AA women (A, B) and EA women (C, D).** Data shown are mean ± SEM from 2-way ANOVA (**A, C**), and as individual points (**B, D**); some points reflect data from more than one individual. Histograms with different lower-case letters differ significantly (*P* < 0.05). The model for AA women indicated a significant main effect of S_I_ (*P* < 0.05), and an S_I_ x GL interaction of *P* = 0.086. No significant main effects or interactions were observed for analyses within EA women.

**Figure 4:**
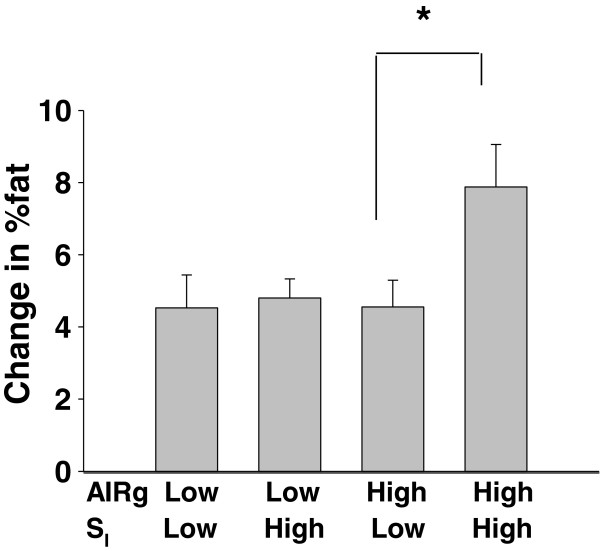
**Change in %fat over 1 year within subgroups stratified by median AIRg and median S**_**I**_**; data adjusted for GL**. Only within women with high AIRg was insulin sensitivity significantly associated with change in %fat.

The ANOVA model for AA women indicated a significant main effect of S_I_ (*P* < 0.05), as well as an S_I_ x GL interaction of *P* = 0.086 (Figure 3A). Within AA women, those who were relatively insulin sensitive and who consumed a relatively high GL diet gained or tended to gain greater %fat than other subgroups (*P* < 0.05 vs both Low S_I_ groups, and *P* = 0.068 vs High S_I_, Low GL). Neither S_I_ nor GL were significant for EA women (Figure 3B). When data were stratified by high vs low AIRg, S_I_ was a significant predictor of change in %fat only in women with high AIRg (Figure 4).

## Discussion

The major conclusion of this study is that within AA but not EA women, obesity predisposition appears related to insulin sensitivity, and that this predisposition is exacerbated by a high insulin response. This conclusion is based in part on the observation that lean, formerly overweight (obesity-prone) AA but not EA women were significantly more insulin-sensitive than their never-obese counterparts. We also found that changes in adiposity over 1 year were related to insulin sensitivity in AA but not EA women, and in women with high but not low insulin response.

Although we cannot determine from this study why obesity predisposition was associated with insulin sensitivity in AA and not EA, this ethnic/race difference likely relates to greater insulin responsiveness in AA. The provocative work of Ludwig and colleagues has shown that serum insulin concentration at 30 minutes after an oral glucose load is a significant predictor of weight gain or loss in certain individuals [12,14]. Numerous studies have shown that healthy AA relative to EA have up to a 2-fold greater insulin response [2-6]. Similarly in this study, AA women had significantly greater AIRg than EA women. Thus, we speculated that the higher AIRg within the AA women may have explained the race-specific results concerning the association of S_I_ with change in adiposity. This hypothesis was supported by the observation that, when analyses were stratified by median AIRg (high/low), an association of S_I_ with change in %fat was observed only within those women with high AIRg. The physiological basis for greater insulin responsiveness in AA women is not clear. Possible explanations include estradiol, which is greater in AA vs EA [29], is associated with AIRg, and promotes beta-cell survival [30]. Alternatively, reactive oxygen species, which are reported to be higher in AA [31] and which play an integral role in insulin secretion [32] may contribute to greater AIRg in AA. Further research is warranted to understand the basis for greater insulin responsiveness in AA.

In previous studies, the association of insulin response with weight gain or loss was observed only within individuals who consumed a relatively high GL diet [12,14]. Thus, we also examined whether dietary GL was associated with change in %fat over 1 year within AA and EA, groups that differ dramatically in their insulin responsiveness. Results indicated that AA women appeared uniquely sensitive to diet quality regarding change in adiposity, an effect that was modulated by S_I_. Only within those AA women who were relatively insulin sensitive did dietary GL appear to influence change in %body fat. Although this observation only approached significance (*P* = 0.086), it supports the hypothesis that higher dietary GL and greater insulin sensitivity synergize to promote adiposity in AA women, who are uniquely sensitive to these factors due to their greater insulin responsiveness. Confirmation of this observation may be important, as it has implications for development of dietary strategies to minimize weight gain or promote weight loss in AA women.

In the broader context of metabolic health, low insulin sensitivity, or insulin resistance, is considered to be an unfavorable condition due to its association with disease risk [33]. Paradoxically, our data and others’ [15,16] suggest that in some cases insulin resistance may protect against obesity. These discrepant observations may imply that the association between insulin resistance and metabolic disease is confounded by weight gain, ectopic fat accumulation, inactivity, and poor diet, factors that may lead to both insulin resistance and disease. In other words, insulin resistance in the context of chronic metabolic disease may, to some extent, be a marker for positive energy imbalance and disease risk-promoting lifestyle. However it is also possible that low S_I_ is a double-edged sword, conferring both leanness and risk for chronic metabolic disease. We cannot determine from this study whether the “protective” aspect of low S_I_ regarding adiposity is beneficial to AA women regarding long-term health. Obesity is not as tightly associated with morbidity and mortality in AA as in EA [34,35]. In contrast, AA are disproportionately burdened by type 2 diabetes [36], a disease that results in part from insulin resistance [37]. Further study is needed to determine if lower insulin sensitivity in AA women is associated with greater risk for type 2 diabetes, even in the absence of obesity.

Strengths of this study were the longitudinal study design, the use of the weight-reduced model, and characterization of insulin sensitivity using IVGTT. Limitations were the convenience sample of volunteers, which may not have been representative of, or generalizable to, the larger population, and the use of self-reported diet; however, reporting bias was similar in AA and EA women [28].

## Conclusions

Lean, formerly overweight (obesity-prone) AA women were more insulin sensitive than their never overweight counterparts. Further, greater S_I_ predicted an increase in %fat over 1 year only in women who were AA or had high AIRg. Dietary GL tended to interact with S_I_ to predict %fat gain in AA. Taken together, these findings suggest that insulin sensitivity, insulin responsiveness, and dietary GL interact to affect adiposity. Insulin sensitive AA women may be uniquely prone to adiposity due to their greater insulin responsiveness. Whether dietary strategies can be indentified to prevent or reverse obesity in AA warrants future study.

## Authors’ contributions

BG provided study oversight, data analysis, and manuscript preparation; JA and NB provided diet analysis and assistance with manuscript preparation and editing; GH provided study design and implementation, and assisted with manuscript preparation. All authors read and approved the final manuscript.
